# Drivers of understory shrub and herb biomass in subtropical Chinese fir forests: a cluster analysis and SEM approach

**DOI:** 10.3389/fpls.2026.1933222

**Published:** 2026-09-03

**Authors:** Duoyi Zhang, Xinhua Huang, Manni Hu, Wende Yan, Xiaoyu Cao

**Affiliations:** 1 Faculty of Forestry, Central South University of Forestry and Technology, Changsha, China; 2 School of Ecology and Environment, Central South University of Forestry and Technology, Changsha, China; 3 National Engineering Laboratory of Applied Technology for Forestry and Ecology in Southern China, Changsha, China; 4 Key Laboratory of State Forestry Administration on Forest Resources Management and Monitoring in Southern Area, Changsha, China

**Keywords:** Chinese fir forest, environmental factors, herb biomass, shrub biomass, stand structure

## Abstract

Understory vegetation plays a key role in forest ecosystem material cycling and energy flow, and studying its biomass dynamics and underlying driving mechanisms is vital for understanding forest ecosystem functions and achieving precise management. In order to elucidate the complex pathways through which stand structure and environmental factors influence understory vegetation biomass, a study was conducted on 180 plots of Chinese fir forests at different developmental stages in the subtropical region of China. The study included 36 plots each of young forest (6 years), middle-age forest (12 years), near-mature forest (20 years), mature forest (25 years), and over-mature forest (30 years) stands. Based on the quantification of shrub and herb biomass in Chinese fir forests and functional clustering, structural equation modeling (SEM) was employed to systematically analyze the direct and indirect effects of environmental factors and stand structure on understory vegetation biomass. The results indicate the following: First, K-means clustering analysis revealed that shrub can be classified into two categories: Tall, dense shrubs (TD-SB) and Low-Sparse Shrubs (LS-SB), and herb can be classified into three categories: Tall, dense herbs (TD-HB), Low-Sparse herbs (LS-HB), and Medium-density herbs (MD-HB); Second, stand structure and environmental factors have significant effects on understory shrub and herb biomass. The Mingling Index of tree species within stand structure exerts the greatest negative effect on shrub biomass and the greatest positive effect on herb biomass. Among environmental factors, slope position and slope gradient have been found to exert a relatively strong positive influence on understory vegetation biomass. The results revealed that all stand structure indicators had a negative effect on the shrub biomass of TD-SB and LS-SB, while environmental factors had a negative impact on MD-HB. This study establishes a theoretical framework for the sustainable management of subtropical Chinese fir forest ecosystems and demonstrates its practical application.

## 1 Introduction

Forest ecosystems are of critical importance to terrestrial ecosystems, playing an indispensable role in global carbon cycling, biodiversity maintenance, and climate regulation (Franklin et al., 2002; Pan et al., 2013). Currently, the intensification of global climate change and increased human disturbance pose dual challenges to the structural integrity and functional stability of forest ecosystems. Quantifying the influence of forest structure on ecological processes and enhancing ecosystem service functions scientifically represent central issues in forestry research (Labarre et al., 2025; Lindner et al., 2010; Seidl et al., 2017). Understory vegetation, as a key component of forest ecosystems, comprises shrubs, herbs, and saplings. It plays an irreplaceable ecological role in maintaining biodiversity, promoting nutrient cycling and soil improvement, and driving the natural regeneration and succession of forest stands (Díaz et al., 2007; Gilliam, 2007; Nilsson and Wardle, 2005; Xiang et al., 2025). Understory vegetation biomass serves not only as a core indicator for evaluating understory productivity, but also profoundly influences stand-level carbon cycling processes and carbon storage capacity through litter decomposition and root turnover (Bello et al., 2010; Kodero et al., 2024). In subtropical regions, understory vegetation contributes nearly 30% of the total net primary productivity in forest ecosystems. Its dynamics exerts a significant regulatory effect on regional carbon balance (Yang et al., 2011). Consequently, the systematic elucidation of the driving mechanisms of understory vegetation biomass in subtropical regions holds considerable theoretical value and practical relevance for predicting the dynamic responses of forest ecosystems to environmental changes and for formulating adaptive management strategies (Alonso-Rodríguez et al., 2022; Wu et al., 2025).

Existing research indicates that understory vegetation biomass is influenced by the combined effects of stand structure and environmental factors. Furthermore, these factors do not act independently; rather, they form a complex network of influences comprising both direct and indirect pathways (Zheng et al., 2025). Conventional statistical methodologies encounter certain challenges in addressing intricate causal relationships that involve latent variables. Structural equation modeling (SEM) is a multivariate statistical technique that integrates factor analysis and path analysis. It can simultaneously handle multiple dependent variables and estimate the relationships between latent and observed variables and quantify direct and indirect effects among variables. SEM is advantageous for analyzing intricate network relationships among multiple variables and testing theoretical hypotheses. It is particularly suitable for the analysis of multilevel, multipath effects of stand structure and environmental factors on understory vegetation biomass (Chen et al., 2025; Uhl et al., 2024). Concurrently, extant studies frequently analyze understory vegetation in its totality, with limited attention to the potential discrepancies in response mechanisms between the shrub and herbaceous layers (Yu et al., 2022). However, extant evidence indicates that shrubs and herbs exhibit significant differences in their utilization thresholds of key resources (e.g., light and nutrients), and these differences may profoundly affect the community assembly process (Shi et al., 2024; Zheng et al., 2025). Therefore, by quantifying understory vegetation biomass, performing cluster analysis, and using SEM to analyze stand structure and environmental factors, we can precisely identify the primary factors influencing understory vegetation biomass in different forest types and elucidate the specific response mechanisms of different types of understory vegetation biomass to these driving factors.

The Chinese fir forests (*Cunninghamia lanceolata* (Lamb.) Hook.), an important fast-growing timber species in China’s subtropical regions, is characterized by rapid growth, high-quality wood, and significant economic value. It is one of the tree species with the largest cultivated area and the highest timber volume in the planted forests of southern China (Farooq et al., 2019; Tian et al., 2011). Chinese fir forests play an irreplaceable role in timber production, carbon sink, and ecological restoration. However, issues such as soil degradation and understory vegetation decline resulting from long-term continuous planting have become increasingly prominent (Bai et al., 2020; Farooq et al., 2019; Tian et al., 2011). Therefore, utilizing Chinese fir forests as a study subject to systematically analyze the mechanisms by which stand structure and environmental factors influence understory vegetation biomass is not only of typical regional representativeness and practical significance but also deepens our understanding of the driving mechanisms of stand structure and environmental factors on understory vegetation biomass. This, in turn, promotes the transformation of understory vegetation management of Chinese fir forests toward precision and facilitates the formulation of sustainable management strategies, and provides important scientific evidence and practical value for the protection of biological diversity (Xu et al., 2022). The objectives of this study are as follows:

1. To quantitatively analyze the patterns of stand structure variation across age classes in Chinese fir forests.
2. To estimate understory shrub and herb biomass in different age classes using allometric equations, and further identify functional groups of understory shrub and herb via K-means clustering.
3. To construct and fit structural equation models to elucidate the direct and indirect pathways through which stand structure and environmental factors influence understory shrub and herb biomass. Based on the model results, we systematically reveals the specific response mechanisms of different types of shrub biomass and herb biomass to driving factors.

## 2 Materials and methods

### 2.1 Study area

**Figure 1:**
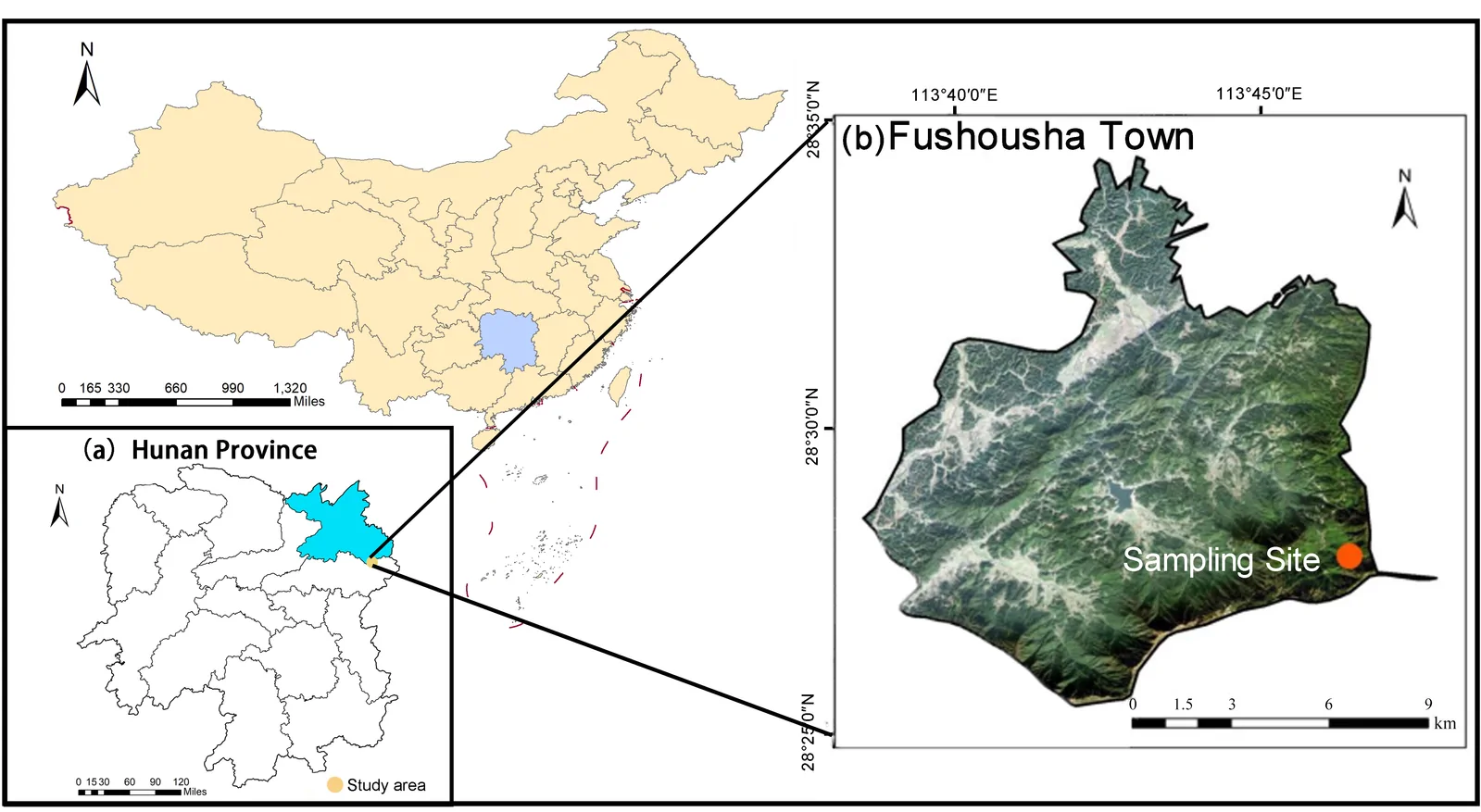
Location map of the study area.

The Fushoulin Site is located in the southern portion of Pingjiang County, Yueyang City, Hunan Province, spanning geographical coordinates ranging from 28°03′00″N to 28°32′30″N and 113°41′15″E to 113°45′00″E (**Figure 1**). The site is located in the Lianyun Mountain branch of the Luoxiao Mountain Range, covering an area of approximately 1,134 hectares. The topography of the study area is characterized by undulating hills, with an overall north-low, south-high elevation gradient ranging from 835 to 1573.3 meters. The region’s climate is typified by a subtropical humid monsoon climate, with marked seasonal variations, with an annual average temperature of 12.1 °C and annual average precipitation of 2100 mm. The region is characterized by yellow-brown soil, typified by its deep profiles and abundant nutrient reserves. The accumulation of rich humus beneath the forest canopy fosters optimal site conditions, conducive to the growth of a diverse array of tree species. The forestry farm is located in the subtropical evergreen broadleaf forest vegetation zone, which is characterized by a high degree of biodiversity and a wealth of natural resources. The most prevalent tree species on the farm are Chinese fir (*Cunninghamia lanceolata* (Lamb.) Hook.) and Huangshan pine (*Pinus hwangshanensis* W. Y. Hsia). The understory shrubs include Chinese sumac (*Rhus chinensis* Mill.), wild prickly ash (*Zanthoxylum simulans* Hance), and beautiful lespedeza (*Lespedeza formosa* (Vogel) Koehne). The key understory herbaceous species comprise Chinese silver grass (*Miscanthus sinensis* Andersson) and broad-leaf (*Indocalamus tessellatus* (Munro) Keng f.).

### 2.2 Experimental design and data collection

**Figure 2:**
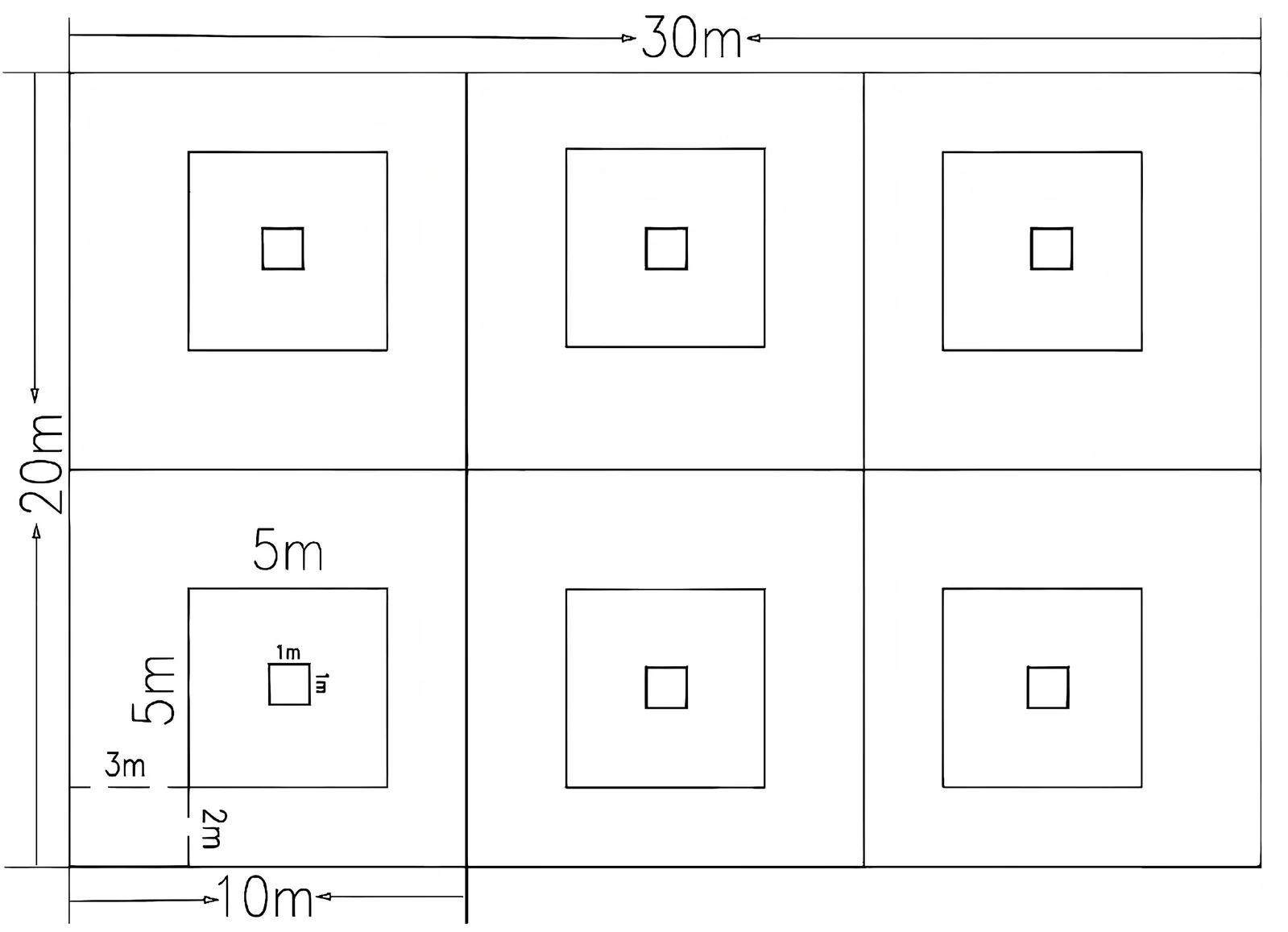
Plot setup diagram.

The classification of Chinese fir stands was primarily based on age, resulting in five age classes: young-aged forest (6 years, YF), middle-aged forest (12 years, MidF), near-mature forest (20 years, NMF), mature forest (25 years, MatF), and overmature forest (30 years, OMF). Six independent replicate plots, each measuring 30 m × 20 m, were established for each age group, amounting to a total of 30 plots. Each plot was further subdivided into six subplots measuring 10 m × 10 m for tree surveys (**Figure 2**). A 5 m × 5 m shrub plot was established at the center of each subplot, positioned 2 m and 3 m from the two plot edges, respectively. Concurrently, a 1 m × 1 m herbaceous plot was established at the center of each shrub plot. Therefore, each age group comprised 36 shrub and herb sample plots (6 main plots × 6 subplots), giving a total of 180 tree-shrub-herb sample plots (5 age groups × 6 main plots × 6 subplots) for the entire study. A total of 8,781 shrubs (147 species from 38 families and 74 genera) and 11,565 herbaceous plants (78 species from 40 families and 62 genera) were recorded within these plots. The biomass of each plant was estimated, and structural equation modeling was subsequently conducted based on the average biomass of shrubs and herbs across 180 plots, along with stand structure indicators, and environmental factors.

The fundamental properties of the sample plots are summarized in **Table 1**. The plot survey content consisted of three categories. The first category included slope gradient (SG), slope position (SP), human disturbance intensity (HDI), and humus thickness (HS). The second category involves basic characteristics of the tree layer, namely tree species, coordinates, diameter at breast height (DBH), tree height, and crown spread for each tree. The third involved collecting basic information on the understory shrub and herb layer, including species names, number of individuals, height, cover, and crown spread volume projection for shrubs and herbs. Furthermore, the presence of saplings with a height of ≥3 m and a DBH of <5 cm was also documented.

**Table 1:** Basic information of plot.

Age class	Age (years)	Average DBH (cm)	Average tree height (m)	Breast height (DBH)	SG (°)	SP	HS (cm)	HDI
YF	6	3.1	2.3	1.1 x 1.1	4-32	All	3-7	From None to Moderate
MidF	12	9.8	6.1	2.3 x 2.3	10-50	Medium	4-8	Light
NMF	20	10.2	7.5	2.4 x 2.7	20-31	Medium	2	Light
MatF	25	13.3	9.8	3.2 x 3.1	40-65	All	8	From Moderate to Intensity
OMF	30	13.9	12	3.2 x 3.3	0-55	Upper, Middle	8-10	From Mild to Intense

“All” represents the presence of upper, middle, and lower slope positions. The intensity of human disturbance was categorized into four levels, as determined by field surveys: none (no tending or harvesting), light (slight human disturbance, with well-preserved understory vegetation), moderate (low frequency of human activity, not severe disturbance, with slight trampling traces in the understory vegetation), and intense (obvious human damage, with severely degraded understory vegetation).

### 2.3 Variable selection and calculation

#### 2.3.1 Stand structure indicators

To quantify stand structure, six stand structural indicators were selected for analysis (Kint et al., 2000; Pielou, 1966; Simpson, 1949). Equations 1–6 are as follows:

Canopy openness(B*_i_*), which reflects spatial transparency:

(1) $$B_{i}=\frac{1}{n}\sum_{j=1}^{n}\frac{D_{ij}}{H_{ij}}$$

Where D*_ij_* is the distance between object tree *i* and adjacent tree *j*, H*_ij_* is the height of adjacent tree *j*.

Mingling index of tree species(H*_i_*), which characterizes the degree of tree species mixing:

(2) $$H_{i}=\frac{n_{i}-k}{16}$$

Where *n_i_* is the count of unique structural units, *k* is the count of differences among tree species.

Simpson’s diversity index(D), which is a composite measure of species richness and evenness:

(3) $$D=1-\sum_{i=1}^{S}P_{i}^{2}$$

Where *S* is the number of tree species present within the designated sample plot, *P_i_* is defined as the ratio of the number of trees of species *i* to the total number of trees.

Pielou’s evenness index(E), which indicates uniformity of species distribution:

(4) $$E=\frac{H}{\ln S}$$

Where *S* denotes the number of tree species present within the designated sample plot, H is the Shannon-Wiener diversity index.

Gini coefficient(GC), which reflects unevenness in tree size distribution:

(5) $$GC=\frac{\sum_{i=1}^{n}(2i-n-1)BA_{i}}{\sum_{i=1}^{n}BA_{i}(n-1)}$$

Where n is the quantity of trees within the designated plot, *BA_i_* is the cross-sectional area of an individual tree.

Coefficient of variation of tree size(cv), which quantifies the dispersion of individual tree sizes:

(6) $$cv=\frac{\sqrt{\frac{\sum_{i=1}^{n}{\left(DBH_{i}-\bar{DBH}\right)}^{2}}{n-1}}}{\bar{DBH}}$$

Where n is the number of trees within the specified sample plot, DBH signifies the arithmetic mean of tree diameter at breast height.

#### 2.3.2 Environmental indicators

The present study selected slope gradient, aspect, humus thickness, and human disturbance intensity as core environmental factors. Although the environmental characteristics of the study plots are closely associated with factors such as climate, soil, and human disturbance, the research area is located within the same forest farm and thus boasts relatively uniform climatic conditions. And since soil conditions varied only slightly, soil factors were not included in the study. Additionally, all plots in the study area were located on the same mountain, and the internal elevation gradient was insufficient to drive the differentiation of water and heat patterns; therefore, elevation and aspect were not included in the analysis. Instead, slope position and slope gradient—which are more sensitive to microenvironmental heterogeneity—were prioritized as topographic factors (Zheng et al., 2025). Furthermore, the thickness of humus exerts a direct influence on the accumulation process of understory vegetation biomass. Additionally, human disturbance serves as pivotal factor driving biomass variations.

#### 2.3.3 Quantification and clustering of understory shrub and herb biomass

The present study is predicated on extant research models and incorporates field survey data, with a biomass model constructed for understory shrubs that incorporates the projected area of the plant (Ac, m²), the projected volume of the canopy (Vc, m³), the basal diameter (D, cm), the plant height (H, m), and the canopy cover (G, %). First, the number of plant individuals in the shrub layer and herb layer within the sample plots was ranked by abundance. The species accounting for >80% of the cumulative plant abundance were identified, and their biomass models were constructed. The methodology of Xie et al. (2018) was adopted for the shrub layer biomass model. For the families contributing to the top 70% of cumulative plant abundance, the biomass model of their dominant species was adopted. For the remaining families, a generic shrub biomass model was applied. The biomass models for understory shrubs are presented in **Table 2**.

**Table 2:** Shrub biomass calculation model.

Family	Dominant species	Model	R^2^	RE
*REERosaceae*	*Spiraea japonica*	*W* = 2.3331*V_c_^1^*^.279^	0.803	–
*Anacardiaceae*	*Rhus chinensis*	*W*= (3.493 + 74.514*A_c_* + 41.635*A_c_*)/1000	–	–
*Fagaceae*	*Quercus fabri*	W=0.5906*V_c_*^0.797^	0.948	–
*Fabaceae*	*Lespedeza formosa*	*W*=(2.17 + 9.462*A_c_* + 266.309*A_c_*)/1000	–	–
*Hamamelidaceae*	*Loropetalum chinense*	W=0.1417*Vc*^0.689^	–	–
*Rubiaceae*	*Gardenia jasminoides*	*W*=(1.888 + 73.454*A_c_* + 144.254*A_c_*)/1000	–	–
*Aquifoliaceae*	*Ilex pubescens*	*W* = 0.8360*A_c_*^0.866^	0.877	0.0179
*Rutaceae*	*Zanthoxylum simulans*	*W* = 0.649(*D*^2^*H*)^0.865^	0.925	–
*Ericaceae*	*Rhododendron simsii*	*W*=(-177.304 + 501.912*H*)/1000	–	–
*Lauraceae*	*Lindera glauca*	*W* = 0.061(*D*^2^*H*)0.915	0.906	0.061
*Theaceae*	*Camellia oleifera*	*W* = 0.0780(*D*^2^*H*)0.934	0.931	0.036
Other Families	–	*W* = 0.1655*V_c_*^0.817^	–	–

W is shrub biomass (kg), Ac is plant projection area (m²), Vc is crown projection volume (m³), D is basal diameter (cm); H is plant height (m).

For the herb layer, families accounting for the top 80% of individual abundance utilized specific biomass models. For the *Poaceae*, *Asteraceae*, and *Cyperaceae* families, with plant heights ranging from 5 to 80 centimeters, the general models described in the book by MEE (2020) were referenced. The families *Pteridaceae*, *Gleicheniaceae*, and *Polypodiaceae* used a mixed-species model; the *Urticaceae* used the biomass model of Meehania urticifolia; the *Asparagaceae* used the biomass model of Maianthemum japonicum; the *Polygalaceae* referred to the empirical model by Gan et al. (2017); and the remaining species used the general model (**Table 3**).

**Table 3:** Herb biomass calculation model.

Family	Dominant species	Model	R^2^	SE	Source
*Poaceae*	*Miscanthus sinensis*	*M* = 0.054920 *H*^0.8030^ *G*^1.0877^	-	-	(MEE, 2020)
*Asteraceae*	*Eupatorium japonicum*	*M* = 0.054920 *H*^0.8030^ *G*^1.0877^	-	-	
*Cyperaceae*	*Carex cruciata*	*M* = -0.054920 *H*^0.8030^ *G*^1.0877^	-	-	
*Pteridaceae*	*Pteridium aquilinum*	*M* = -0.017*H*^2^+1.01*H*^-6.707^	0.899	0.174	(Sun et al., 2017)
*Gleicheniaceae*	*Dicranopteris dichotoma*	*M* = 0.0222*H*^1.324^	0.780	0.461	
*Polypodiaceae*	*Microsorum fortunei*	*M* = 0.0222*H*^1.324^	0.780	0.461	
*Asparagaceae*	*Liriope spicata*	*M* = 0.008*H*^1.805^	0.985	0.157	(Sun et al., 2017)
*Selaginellaceae*	*Selaginella uncinata*	*M* = 0.0222*H*^1.324^	0.780	0.461	
*Valerianaceae*	*Patrinia scabiosifolia*	*M* = 0.0222*H*^1.324^	0.780	0.461	
*Urticaceae*	*Urtica macrorrhiza*	*M* = 0.0536*H*^1.009^	0.780	0.461	
*Polygalaceae*	*Polygala hongkongensis*	*M* = 0.1128(*HG*)^1.471231^	-	-	(Gan et al., 2017)
Other families	-	*M* = 0.028*H*^2^+1.503*H* + 0.074	0.655	4.092	(Bai, 2023)

M is herb biomass (t·hm^-2^), G is coverage (%), H is plant height (m).

After biomass was estimated using the models, K-means clustering was applied to the data to better elucidate distribution patterns. The Elbow Method and Silhouette Coefficient were then used to determine the optimal number of clusters (K value). The Elbow Method is a heuristic commonly used to determine the optimal K value in clustering algorithms such as K-means. The core idea is to observe how the “loss function” (sum of squared errors, SSE) changes under different K values and to select the point at which the rate of decrease slows sharply at the optimal K value. The calculation formula is provided in Equation 7. Additionally, the silhouette coefficient was employed to ascertain a more reliable number of clusters; the calculation formula is presented in Equation 8. The silhouette coefficient measures the quality of sample allocation among clusters. With values approaching 1 indicating better clustering performance.

The formula for the Sum of Squared Errors (SSE) is as follows:

(7) $$\text{SSE}={\sum_{\text{i}=1}^{\text{k}}\sum_{\text{x}\in C_{i}}∥\text{x}-\mu_{i}∥}^{2}$$

Where *k* is the number of clusters, *C*_i_ is the sample set of the *i* cluster and *μ_i_* indicates the center of the i cluster, $∥X-\mu_{i}{∥}^{2}$ is the squared distance from sample point *x* to the cluster center.

The silhouette coefficient formula is as follows:

(8) $$\text{Silhouette}=\frac{b(i)-a(i)}{max\{a(i),b(i)\}}$$

Where *a*(*i*)is average distance from sample *i* to other samples in the same cluster (reflecting intra-cluster compactness), *b*(*i*) is the average distance from sample *i* to the nearest other cluster (reflecting inter-cluster separation).

### 2.4 Structural equation model construction

SEM is a multivariate statistical analysis method used to construct, estimate and test causal models. It incorporates regression analysis, factor analysis, path analysis and multivariate analysis of variance. SEM comprises measurement and structural models, the formulas of which are as shown in Equations 9–11:

(9) $$X=\Lambda_{x}\xi +\delta$$

(10) $$Y=\Lambda_{y}\eta +\epsilon$$

(11) η=Bη+Γξ+ζ

Where: X represents the observed variables or measurement indicators of ξ; *Y* represents the observed variables or measurement indicators of η; ${\text{Λ}}_{x}$ denotes the correlation coefficient matrix between X and ξ; ξdenotes the exogenous latent variable; ${\text{Λ}}_{y}$ denotes the correlation coefficient matrix between *Y* and η; η denotes the endogenous latent variable; δ denotes the measurement error of the variable *X*; ϵ denotes the measurement error of the variable *Y*; Γ is the correlation coefficient matrix between ξ and η; *B* is the correlation coefficient matrix among ξ; ξ is the error term for η.

Prior to constructing the structural equation model, this study standardized the raw data using the Z-score method, assessed the reliability of the modeling data using Cronbach’s alpha, and evaluated the validity of the modeling data using the Kaiser-Meyer-Olkin (KMO) index and Bartlett’s sphericity test.

**Figure 3:**
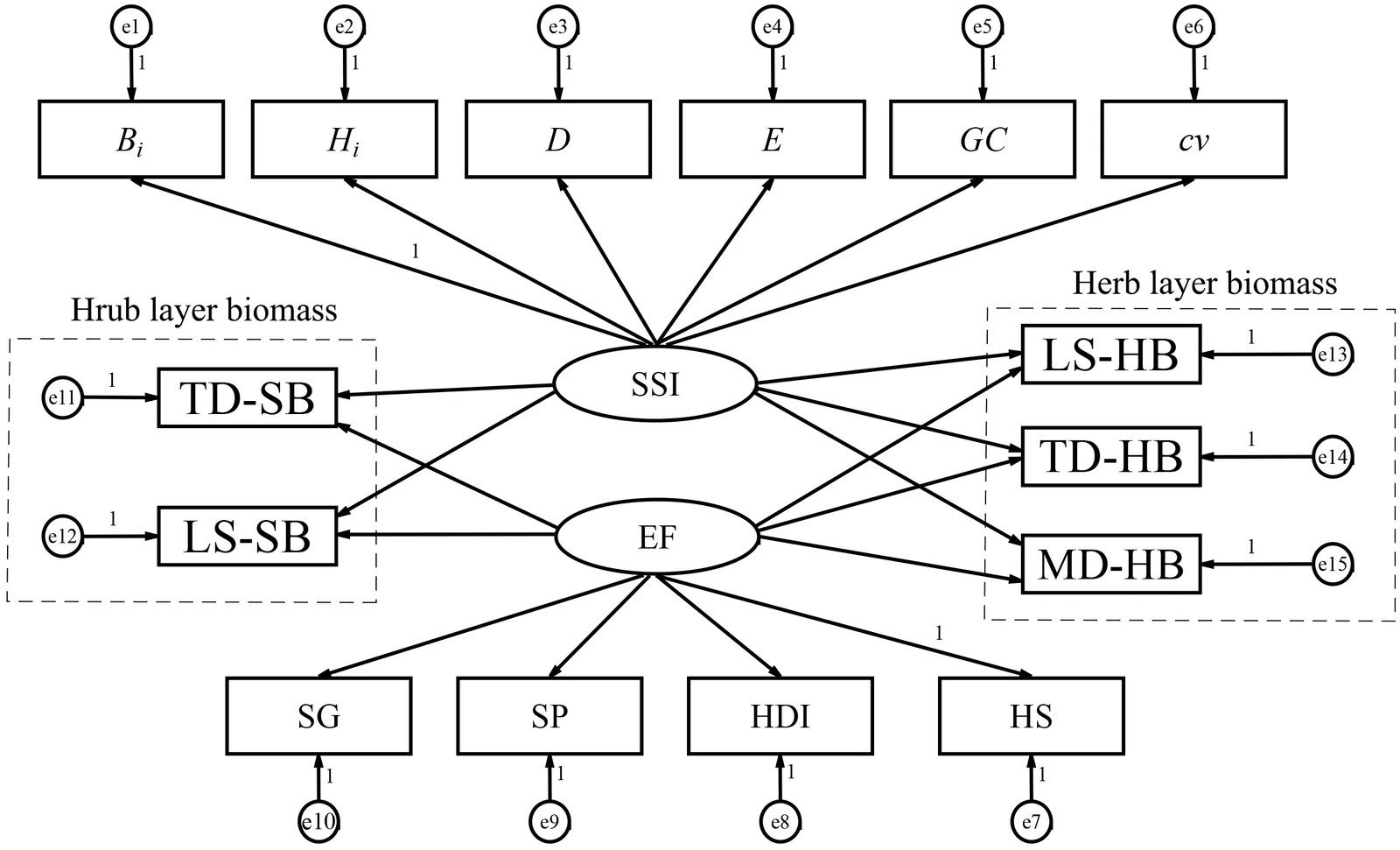
SEM path diagram theoretical indicating influence of environment and stand structure on understory vegetation biomass. SSI is the stand structure index, EF is the environmental factor, and e1-e15 represent the error variables of the model.

The present study considered stand structure (SSI) and environmental factors (EF) as exogenous latent variables. The selected stand structure indicators served as measurable variables for stand structure, namely canopy openness (B), mingling index of tree species (H_i_), Simpson’s diversity index (D), Pielou’s evenness index (E), Gini coefficient (GC), and coefficient of variation of tree size (cv). The slope gradient (SG), slope position (SP), human disturbance intensity (HDI), and humus thickness (HS) were designated as measurable variables for environmental factors. Shrub layer biomass and herb layer biomass were taken as endogenous latent variables. The biomass of tall, dense shrubs (TD-SB) and low, sparse shrubs (LS-SB) was used as the measurable variables for shrub layer biomass. The tall, dense herbs biomass (TD-HB), low, sparse herbs biomass (LS-HB), and medium herbs biomass (MD-HB) served as measurable variables for herb layer biomass. The SEM was constructed to examine the influence pathways of stand structure and environmental factors on understory vegetation biomass. The proposed theoretical model is illustrated in **Figure 3**.

### 2.5 Model fit assessment

This study selected the following indicators for analysis: normed chi-square (CMIN/DF), goodness-of-fit index (GFI), root mean square error of approximation (RMSEA), and incremental fit index (IFI). The suitability of the model was then tested.

### 2.6 Statistical data analysis

The fundamental data organization and calculations for this study were conducted using Microsoft Excel 2016. The statistical analysis was conducted using SPSS 25.0 and R 4.4.2. The construction of the SEM and the presentation of the results were completed using AMOS 24.0. The graphing elements were produced using Origin 2022 and Adobe Photoshop CS6.

## 3 Results

### 3.1 Analysis of stand structure characteristics in Chinese fir forests

**Figure 4:**
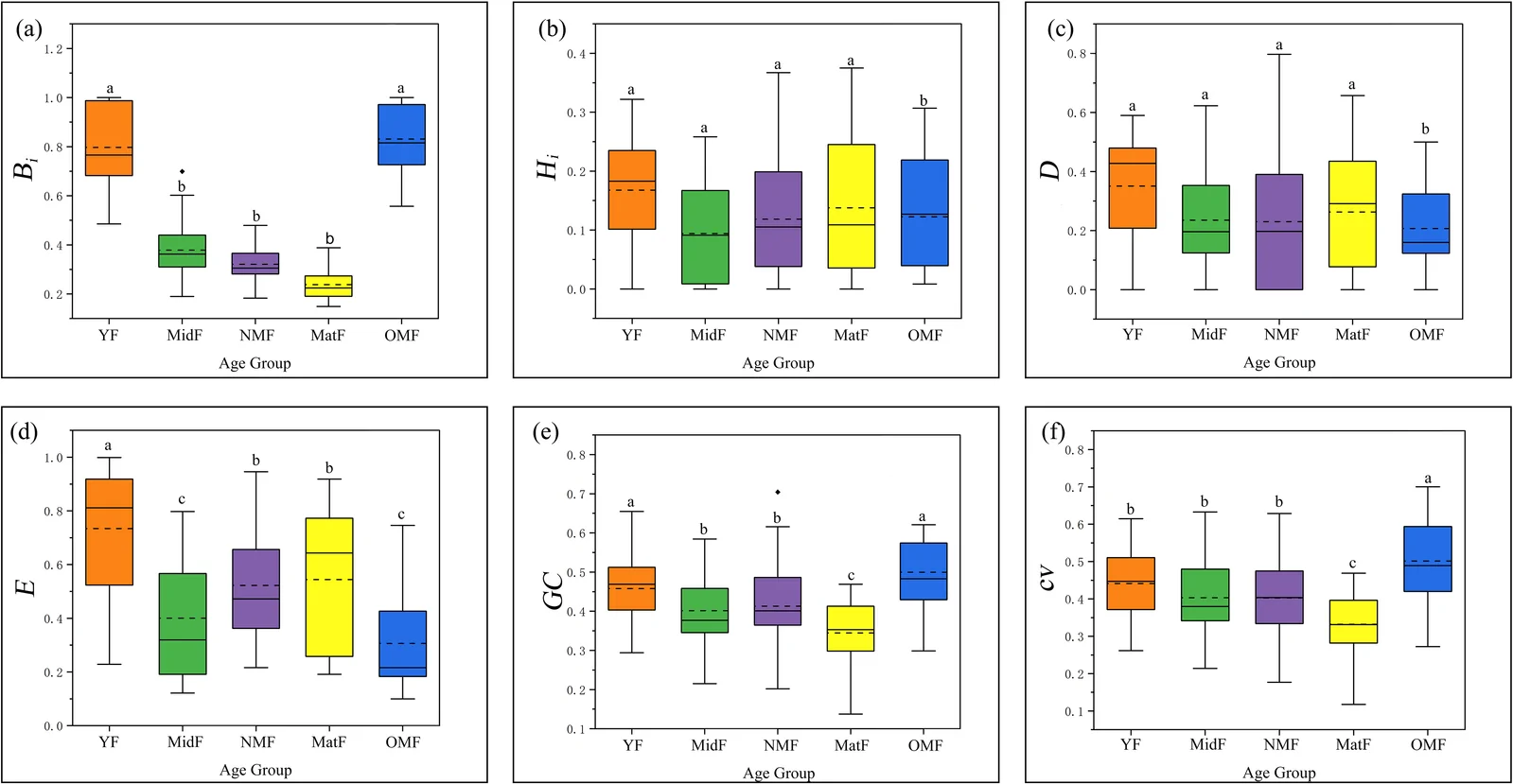
Boxplots of stand structure for age groups of Chinese fir forests: **(a)** canopy openness; **(b)** mingling index of tree species; **(c)** Simpson's diversity index; **(d)** Pielou's evenness index; **(e)** Gini coefficient; **(f)** coefficient of variation of tree size.Note: It is important to note that the use of different letters indicates significant differences (p<0.05) among stand structure indicators of Chinese fir forests across different age groups.

The canopy openness of Chinese fir stands at different age groups exhibited a pattern of first decreasing and then increasing with the growth of stand age (**Figure 4**). It is noteworthy that both young-aged forests and overmature forests exhibited significantly higher levels of openness compared to other age classes (p<0.05). The Mingling Index of tree species is significantly lower in over-mature forests than in other age groups (p<0.05), indicating that diversity first declines and then stabilizes as Chinese fir forest age increases. The diversity of species was found to be significantly lower in over-mature forests in comparison to other age groups (p<0.05). This finding suggests that species diversity tends to decrease as the age of the Chinese fir forests increases. Pielou’s evenness index revealed significant disparities among age groups (p<0.05), with over-mature forests demonstrating the least even distribution. The Gini coefficient and cv reached their peak in over-mature forests, this indicates that the uneven distribution of species and the degree of variation in individual size increase with Chinese fir forest maturity. This combination of “low diversity and high size variation” may be related to the natural thinning and patchy regeneration of over-mature forests. Intensified competition in overmature forests leads to increased natural thinning, causing dominant individuals to grow even larger and facilitating patchy regeneration in canopy gaps, thereby forming a multi-layered structure with significant size differentiation (Zhu et al., 2011). This gap-driven regeneration pattern results in a spatially heterogeneous distribution of individuals across different diameter classes, thereby reducing species diversity while amplifying the degree of variation in individual size.

### 3.2 Biomass measurement and analysis of understory shrub and herb in Chinese fir forests

#### 3.2.1 Statistics and analysis of understory shrub biomass

The elbow method and silhouette coefficient were employed to classify the shrub layer biomass into two clusters. The resulting clustering information for different species within each plot is presented in **Table 4**. TD-SB and LS-SB represent shrub layer biomass cluster 1 and 2, respectively. The classification results suggest that the presence of different clusters may reflect the differential adaptability of plants to varying environmental conditions. The TD-SB shrubs exhibit a mean height of 1.0168 m and an average density of 11 plants per unit area, characterized them as a tall and densely packed type. These shrubs typically have large leaf area and specific leaf area, and they gain a competitive advantage in well-lit environments, such as forest clearings, through rapid growth (Cao et al., 2020). The corresponding mean height of the shrubs in the LS-SB category is 0.5988 meters, and the mean density is 5 plants per unit area; this category can be described as low and sparsely packed. This is characterized by a smaller leaf area, higher leaf tissue density, and higher leaf dry matter content, allowing the plants to adapt to the low-light conditions under the forest canopy through slow growth and nutrient storage (Cao et al., 2020).

**Table 4:** Cluster centers and species composition of shrub layer biomass.

Clustering categories	Average plant height (m)	Average number of plants (plants)	Shrub species	Family
TD-SB	1.0168	11	*Spiraea japonica* L. f.	Rosaceae
			*Rhus chinensis* Mill.	Anacardiaceae
			*Alpinia japonica* (Thunb.) Miq.	Zingiberaceae
			*Symplocos hunanensis* Hand.-Mazz.	Symplocaceae
			*Zanthoxylum simulans* Hance	Rutaceae
			*Lespedeza formosa* (Vogel) Koehne	Fabaceae
LS-SB	0.5988	5	*Loropetalum chinense* (R. Br.) Oliv.	Hamamelidaceae
			*Rhus chinensis* Mill.	Anacardiaceae
			*Spiraea japonica* L. f.	Rosaceae
			*Zanthoxylum simulans* Hance	Rutaceae
			*Camellia oleifera* Abel.	Theaceae
			*Lespedeza formosa* (Vogel) Koehne	Fabaceae

Since the height and number of plants of the same understory vegetation species vary across different plots, shrubs of the same species may be classified into different shrub biomass categories.

**Figure 5:**
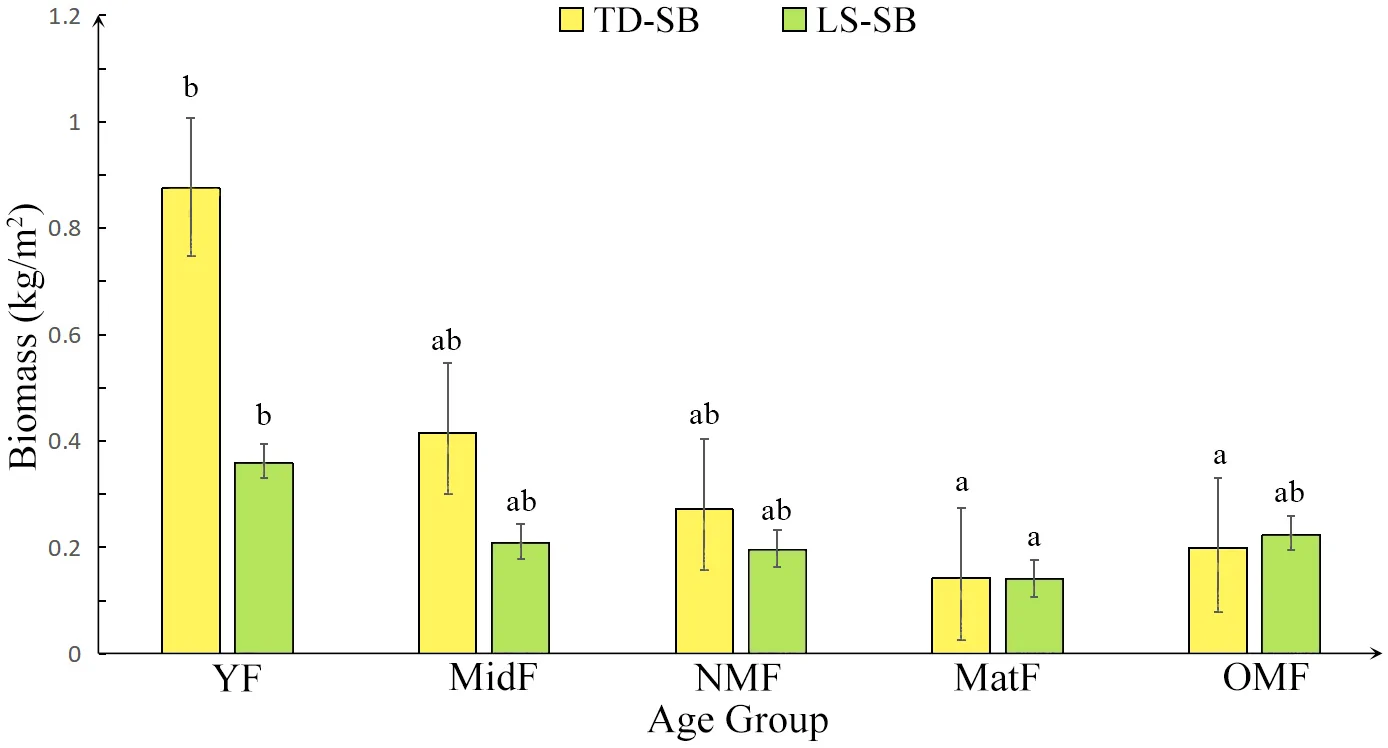
Analysis of shrub biomass among age groups of Chinese fir forests after clustering. Different letters indicate significant differences in biomass among age groups of Chinese fir forests (p<0.05). The error bars in the figure represent the standard error (SE).

The distribution of shrub layer biomass exhibited variability among different age groups in Chinese fir forests; however, it tended to become more uniform as forest age increases, as illustrated in **Figure 5**. The TD-SB (biomass of tall, dense shrubs) and LS-SB (biomass of low, sparse shrubs) exhibited significant disparities across all age classes (p<0.05). In young-aged forests with ample sunlight, TD-SB dominated forest gaps with an absolute advantage of 0.875 kg·m^-2^, while LS-SB yielded only 0.359 kg·m^-2^. As forests transitioned into the middle-aged stage, canopy closure accelerated rapidly, resulting in a significant decrease in TD-SB to 0.415 kg·m^-2^ and LS-SB to 0.208 kg·m^-2^. This maintained a disparity of approximately twofold. As the forest approached maturity, canopy closure continued to increase. The TD-SB further decreased to 0.271 kg·m^-2^, while the LS-SB remained relatively stable at 0.196 kg·m^-2^, narrowing the gap to a factor of 1.4 times. In mature forests with the densest canopy, TD-SB decreased to its lowest value of 0.143 kg·m^-2^, and LS-SB decreased only slightly to 0.143 kg·m^-2^, marking the first time both biomass types were equal. In overmature forests, Lighting conditions improved, and the increase in forest gaps and fallen trees led to a slight recovery of TD-SB to 0.198 kg·m^-2^, while LS-SB increased significantly, reaching 0.223 kg·m^-2^. This finding suggests that late-successional communities are characterized by the predominance of shade-tolerant species regeneration.

#### 3.2.2 Statistic and analysis of understory herbaceous biomass

Based on the elbow method and silhouette coefficient, we divided the herb layer biomass into three clusters, obtaining the detailed clustering information for different species within each plot, presented in **Table 5**. LS-HB, TD-HB, and MD-HB represent herb layer biomass clusters 1, 2, and 3, respectively. The classification results suggest that the presence of different clusters may reflect the differential adaptability of plants to varying environmental conditions. LS-HB denotes the biomass of low-sparse herbaceous plants, exhibiting an average plant height of 0.21 m and the lowest canopy cover (8.62%). These species exhibit an overall low and sparse distribution pattern, contributing limited biomass, and are commonly found in microhabitats characterized by intense sunlight, poor soil, or frequent disturbance. These plants typically have a high specific leaf area and low leaf dry matter content, and they adapt to highly disturbed habitats through rapid growth and reproduction (Ji et al., 2023). TD-HB denotes the total biomass of tall, dense herbaceous plants, exhibiting the maximum plant height (0.51 m) and the highest canopy cover (10.33%). Individuals within this community are characterized by their relatively large stature and dense packing, which confer advantages in light interception and spatial occupation capabilities within their environment. This group is considered the primary contributor to herb layer biomass. They are typically found in forest gaps or edges where moisture and nutrient conditions are favorable. Its greater plant height and canopy cover give it an advantage in competition for light resources, allowing it to occupy an ecological niche by rapidly acquiring resources (Ji et al., 2023). MD-HB denotes the biomass of medium-sized herbaceous plants, exhibiting moderate plant height (0.36 m) but the lowest canopy cover (6.08%). The mean plant height falls between those of the TD-HB and LS-HB groups, yet the community density is sparser than that of the TD-HB group, representing an intermediate state in terms of height and distribution. This pattern has been observed in transitional succession zones or environments experiencing mild stress. Plants of this type have adapted to environments with moderate light levels and fluctuating resources through conservative resource utilization (Ji et al., 2023).

**Table 5:** Cluster centers and species composition of hurb layer biomass.

Clustering categories	Average plant height (m)	Coverage mean	Hurb species	Family
LS-HB	0.2079	8.62%	*Miscanthus sinensis* Andersson	Poaceae
			*Carex cruciate Wahlenb*	Cyperaceae
			*Oplismenus compositus* (L.) P. Beauv	Poaceae
			*Indocalamus tessellatus* (Munro) Keng f	Poaceae
			*Polygala hongkongensis* var. *stenophylla* Migo	Polygalaceae
			*Selaginella uncinata* (Desv.) Spring	Selaginellaceae
TD-HB	0.5101	10.33%	*Miscanthus sinensis* Andersson	Poaceae
			*Oplismenus compositus* (L.) P. Beauv	Poaceae
			*Dicranopteris pedata* (Houtt.) Nakaike	Gleicheniaceae
			*Pteridium aquilinum* var. *latiusculum* (Desv.) Underw. ex Heller	Pteridaceae
			*Liriope spicata* (Thunb.) Lour	Asparagaceae
			*Microsorum fortunei* (T. Moore) C. M. Kuo	Polypodiaceae
MD-HB	0.3566	6.08%	*Indocalamus tessellatus* (Munro) Keng f	Poaceae
			*Miscanthus sinensis* Andersson	Poaceae
			*Microsorum fortune* (T. Moore) C. M. Kuo	Polypodiaceae
			*Patrinia scabiosaefolia* Link	Valerianaceae
			*Selaginella uncinate* (Desv.) Spring	Selaginellaceae
			*Liriope spicata* (Thunb.) Lour	Asparagaceae

Since the height and cover of the same herbs plant may vary across different sample plots, plants of the same species may be classified as different herbs biomass types.

**Figure 6:**
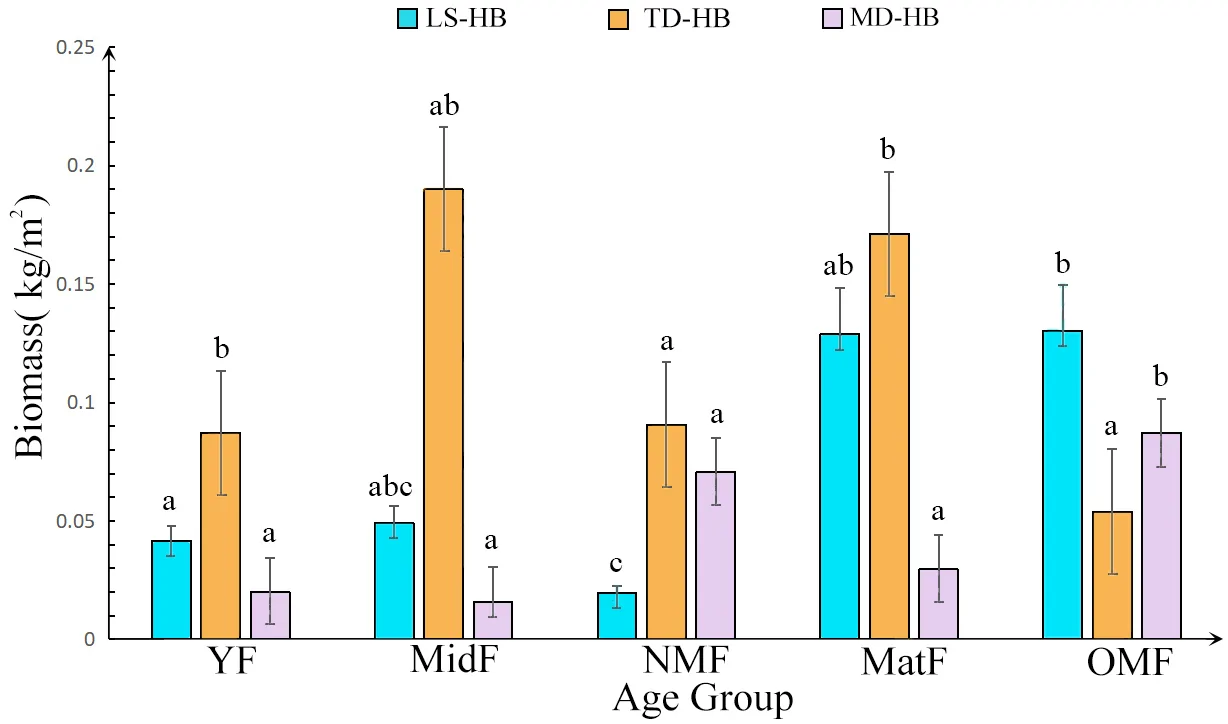
Analysis of herb biomass among age groups of Chinese fir forests after clustering. Different letters indicate significant differences in biomass among age groups of Chinese fir forests (p<0.05). The error bars in the figure represent the standard error (SE).

Significant differences in herb biomass were observed across age groups of Chinese fir forests, as shown in **Figure 6**. In young-aged forest herbaceous communities, biomass was predominantly composed of TD-HB (tall, dense herbaceous biomass), which reaches 0.087 kg·m^-2^, approximately twice that of LS-HB (low-sparse herbaceous biomass), while MD-HB (medium-type herbaceous biomass) accounted for only 0.020 kg·m^-2^. In the middle-aged forest, TD-HB surged to a peak of 0.190 kg·m^-2^, LS-HB rose slightly to 0.049 kg/m², and MD-HB decreased marginally to 0.016 kg·m^-2^. These results indicate that the canopy had not yet fully closed and that sun-loving, low-growing species were rapidly expanding. During the near-mature stage, canopy closure significantly increased, and TD-HB decreased to 0.019 kg/m², while LS-HB increased to 0.091 kg·m^-2^, respectively. Concurrently, the shade-tolerant, medium-sized MD-HB rose to 0.071 kg·m^-2^, thereby becoming the key factor sustaining the herbaceous layer biomass. In mature forests with the densest canopy, TD-HB rebounded to 0.129 kg·m^-2^, LS-HB remained at 0.171 kg·m^-2^, and MD-HB again decreased to 0.030 kg·m^-2^. This finding suggests that forest gaps or microhabitat heterogeneity enabled tall and low-growing species to regain access to resources by tall and low-growing species. In overmature forests, the increased frequency of fallen trees and forest gaps allowed TD-HB to increase slightly to 0.130 kg·m^-2^, thereby maintaining its dominance. The LS-HB exhibited a significant decrease, reaching 0.054 kg·m^-2^, while the MD-HB increased, reaching 0.087 kg·m^-2^. This observation indicates the rapid recovery of tall, dense herbaceous species in response to enhanced light conditions.

### 3.3 Analysis of the impact of stand structure and environment factors on understory shrub and herb biomass of Chinese fir forests

#### 3.3.1 Reliability and validity of modeling data

Reliability and validity tests were conducted for the two exogenous latent variables – environmental factors and stand structure – both of which met the requirements for structural equation modeling (**Table 6**). Initially, it was evident that the KMO sampling adequacy measures were all greater than 0.80 (environmental factors 0.878, stand structure 0.883). Furthermore, the Bartlett’s sphericity test yielded a significant approximate chi-square value (p<0.001). This finding suggests the presence of sufficient correlation among the observed variables, thereby indicating their suitability for factor analysis. Furthermore, Bartlett’s test of sphericity yielded a highly significant approximate chi-square value (p<0.001). This finding suggests the presence of adequate inter-observational correlation among the observed variables, thereby indicating their suitability for factor analysis. Secondly, Cronbach’s α coefficients were 0.882 and 0.883, respectively, both exceeding the 0.70 threshold, indicating good internal consistency reliability. All observed variables significantly contributed to the latent variables, ensuring convergent validity. In summary, the measurement model for environmental factors and stand structure demonstrated adequate reliability and validity, thus paving the way for further analysis through exploratory factor analysis or comprehensive SEM estimation.

**Table 6:** Reliability test and validity test results for modeling data.

Latent variable	Observed variable	Cronbach’s alpha	KMO’s test and Bartlett’s test		
Environmental factors	Slope gradient	0.882	KMO Sampling Adequacy Measure		0.878
	Slope position		Bartlett’s test of sphericity	Approximate chi-square	35.215
	Human disturbance intensity			DF	6
	Humus thickness			Significance	0
Stand structure	Canopy openness	0.883	KMO Sampling Adequacy Measure		0.744
	Mingling index of tree species				
	Simpson’s diversity index		Bartlett’s test of sphericity	Approximate chi-square	1120.92
	Pielou’s evenness index			DF	15
	Gini coefficient			Significance	0.000
	Coefficient of variation of tree size		–	–	–

The structural equation modeling results showed that the Chi/DF ratio was 1.166, the model’s GFI was 0.987, and the Adjusted GFI (AGFI) was 0.956,both exceeding 0.9. The RMSEA value was 0.030, which is below 0.08 and indicates a favorable model-data fit.

#### 3.3.2 Analysis of the mechanisms by which environment and structure affect understory vegetation biomass

**Figure 7:**
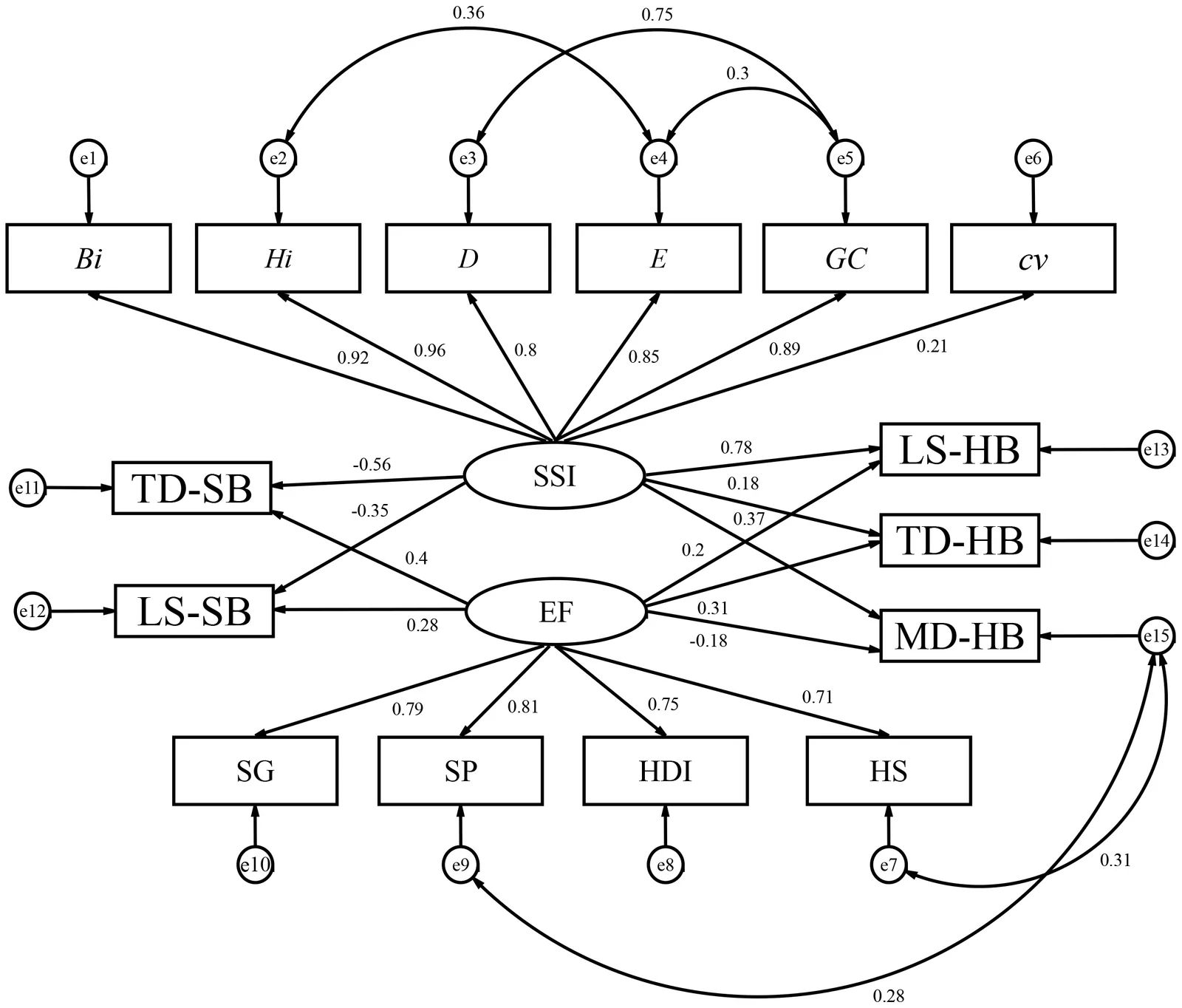
SEM path diagram of the influence of stand structure and environment on understory shrub and herb biomass.

The standardized structural path coefficients of the model are shown in **Figure 7**. The impact of stand structure and environmental factors on herb and shrub biomass are different; stand structure exerts a more substantial influence. The primary factors affecting stand structure are canopy openness, mixture degree, Gini coefficient, Pielou’s evenness index, and D, while slope gradient and slope position are key environmental factors. These factors act together on the distribution and growth of understory vegetation biomass.

The results of the structural equation model show a clear differentiation between the direct and indirect effects of stand structure and environmental factors on shrub and herb biomass. Standardized path coefficients indicate that stand structure has a strong direct negative effect on shrub biomass (-0.28 to -0.54), while its indirect positive effect on herb biomass is more pronounced (0.14 to 0.75); Environmental factors exerted a direct positive effect on the biomass of both shrub types ranging from 0.20 to 0.32, while they exhibited a weak negative effect on MD-HB (-0.13 to -0.15).

The direct effects and indirect effects of environmental factors and forest stand structure on understory shrub and herb biomass of Chinese fir forests are demonstrated in **Table 7**. Environmental factors including slope gradient, slope position, human disturbance intensity, and humus thickness all exerted positive effects on both shrub biomass types (TD-SB and LS-SB). This finding suggests that environments characterized by more complex topography, stronger human disturbance intensity, and richer soil nutrients are more conducive to shrub biomass accumulation. Moreover environmental factors exhibited a positive correlation with both TD-HB and LS-HB, though with relatively weaker effects. In contrast, the MD-HB exhibited a slight negative effect on understory herb biomass, suggesting that medium-density herbs prefer relatively stable habitat conditions. This difference may stem from the fact that the species to which MD-HB belongs possesses conservative functional traits, such as high leaf dry matter content and low specific leaf area, which result in a narrower tolerance range for resource fluctuations and make the species more susceptible to growth inhibition when nutrient or water conditions change (Cao et al., 2020).

**Table 7:** Direct effects and indirect effects of stand structure and environmental factors on understory vegetation biomass.

Indicator		Shrub layer					Herb layer					
		TD-SB			LS-SB		LS-HB		TD-HB		MD-HB	
		Direct effects		Indirect effects	Direct effects	Indirect effects	Direct effects	Indirect effects	Direct effects	Indirect effects	Direct effects	Indirect effects
Environmental factors	Slope gradient		0.4^***^	0.32^***^	0.28^**^	0.22^**^	0.2^**^	0.16^*^	0.31^**^	0.24^**^	-0.18^*^	-0.14^*^
	Slope position			0.32^***^		0.23^**^		0.16^*^		0.25^**^		-0.15^*^
	Human disturbance intensity			0.30^***^		0.21^**^		0.15^*^		0.23^**^		-0.14^*^
	Humus thickness			0.28^**^		0.20^**^		0.14^*^		0.22^**^		-0.13^*^
Stand structure	Canopy openness		-0.56^***^	-0.54^***^	-0.35^***^	-0.34^**^	0.78^***^	0.75^***^	0.18^*^	0.17^*^	0.37^***^	0.36^**^
	Mingling index of tree species			-0.52^***^		-0.32^**^		0.72^***^		0.17^*^		0.34^**^
	Simpson’s diversity index			-0.45^***^		-0.28^**^		0.62^***^		0.14^*^		0.30^**^
	Pielou’s evenness index			-0.48^***^		-0.30^**^		0.66^***^		0.15^*^		0.31^**^
	Gini coefficient			-0.50^***^		-0.31^**^		0.69^***^		0.16^*^		0.33^**^
	Coefficient of variation of tree size			-0.52^***^		-0.32^**^		0.72^***^		0.17^*^		0.34^**^

*** indicates p < 0.001, ** indicates p < 0.01, and * indicates p < 0.05. The values represent standardized path coefficients.

All stand structure indicators demonstrated consistent and significant negative impacts on shrub biomass (-0.28 to -0.54). This was particularly evident in the pronounced suppression of TD-SB. This finding suggests that the presence of more complex stand structures, higher canopy openness, and greater tree size differentiation can impede the accumulation of shrub biomass. In contrast, the structural diversity indicators exhibited a positive effect on herb biomass, manifesting distinct type-specific differentiation. Specifically, the LS-HB demonstrated a remarkably robust promotion (0.62 to 0.75); MD-HB exhibited a moderately positive effect (0.30 to 0.36); while TD-HB exhibited only a weak positive correlation (0.14 to 0.17). The findings indicate that stand structure complexity exerts a substantial inhibitory effect on shrub growth by modulating light availability and nutrient competition within the forest ecosystem. Concurrently, these structural elements engender optimal growth conditions for shade-tolerant or sun-loving herbaceous plants, thereby precipitating substantial niche differentiation.

### 3.4 Management enlightenment for plantations of different forest ages

To increase the biomass of understory vegetation, this study proposes a series of optimization measures based on near-natural management principles, tailored to the characteristics of Chinese fir forests at various age classes. For young forests, it is recommended to protect existing shrubs and herbs plants, avoid excessive disturbance, and increase light penetration and soil fertility through moderate thinning and soil management to promote vegetation growth. In the context of middle-aged forests, the optimization of stand structure through thinning and the promotion of mixed forest formation are crucial for enhancing light penetration. Additionally, the implementation of understory vegetation management is imperative to stimulate biomass growth. Optimization measures for near-mature forests include selective cutting and targeted cultivation of specific vegetation, along with soil management to utilize naturally sparse areas and increase diversity. The management practices employed in mature forests emphasize the promotion of regeneration and the renewal of stands, with a focus on the introduction of new tree species while preserving existing mature trees to ensure adequate shade provision. The management of understory vegetation has been demonstrated to enhance soil structure. Overmature forests, on the other hand, require more thorough regeneration, such as clear-cutting or selective cutting, planting a diverse range of tree species and shrubs, implementing soil improvement measures, and establishing ecological corridors to facilitate species migration and gene flow, thereby maximizing the biomass of understory vegetation.

## 4 Discussion

### 4.1 Changes in stand structure with forest age

This study employed a set of six indicators— canopy openness, the mingling index of tree species, Simpson’s diversity index, Pielou’s evenness index, the Gini coefficient, and the coefficient of variation —to comprehensively characterize stand structure. The findings of this study revealed regular changes in forest structure of Chinese fir forests across developmental stages. Furthermore, the co-evolutionary patterns of multiple indicators demonstrated alignment with classical forest succession theory (Oliveras and Malhi, 2016). Among these, canopy openness directly influences understory light conditions and serves as a key driver of understory vegetation growth (Chávez and Macdonald, 2009). Canopy openness exhibits a U-shaped pattern with forest age, with higher values recorded in young and over-mature forests. This finding is fully consistent with the dynamic succession process, wherein the canopy transitions from open to closed and subsequently reopens due to natural disturbances, such as gap formation (Lasky et al., 2015). Concurrently, we observed substantial declines in species diversity (Simpson’s diversity index) and evenness (Pielou’s evenness index) during the over-mature forest stage, accompanied by pronounced increases in dominance (Gini coefficient) and individual size variation (coefficient of variation of tree size). This series of characteristics indicates that in the late succession stage, the community is dominated by a few dominant tree species. Interspecific competition and niche differentiation result in highly heterogeneous population structures and spatial patterns (Zeng et al., 2024). Such stands, distinguished by the presence of concentrated dominant species and complex structures, exhibit the typical features of the climax community or old-growth forest stage (Stoll and Bergius, 2005). In addition, previous studies have demonstrated that stand management has a substantial impact on the growth and metabolism of individual trees. This, in turn, changes the susceptibility of other biotic and abiotic components to disasters (Hervé et al., 2009). This finding indicates that effective stand management practices can regulate and optimize the biomass of understory shrubs and herbs. The present study provides scientific recommendations for stand management strategies in forest operations.

### 4.2 The regular distribution pattern of understory biomass varies with forest age

The present study systematically quantified changes in understory shrub and herb biomass by constructing allometric growth models for shrubs and herbaceous plants in Chinese fir forests, in conjunction with cluster analysis. To this end, this study focuses on the species composition characteristics of the shrub and herbaceous layers. A multifaceted approach was employed, encompassing both universal and species-specific models. This stratified, taxonomic modeling strategy enhances biomass estimation accuracy (Liu et al., 2022). Allometric equations have been extensively adopted by forestry researchers for the estimation of vegetation biomass (Huang et al., 2025; Zhang et al., 2021). This method offers two advantages: it is non-destructive and *in-situ* observation, while avoiding interference with normal plant growth and development (Zhou et al., 2021). This study innovatively combines biomass estimation results with plant morphological indicators (plant height, plant density) in cluster analysis. This integration is motivated by the fact that the biomass of understory vegetation exhibits significant spatial heterogeneity, arising from the complex interactions among microhabitat conditions, forest canopy structure, and interspecific competition (Ali et al., 2020; Markus et al., 2021). Traditional methods for analyzing continuous variables struggle to capture the discontinuous features of biomass distribution (Picard et al., 2021). The findings suggest that the distribution of understory shrub and herb biomass in Chinese fir forests demonstrates regular succession dynamics in relation to forest age (Ares et al., 2010). Specifically, there is a notable shift in the dominance of shrub biomass, with light-demanding tall species progressively giving way to shade-tolerant short species. Additionally, the herb layer exhibits more complex fluctuations, with dominant types alternating in response to canopy opening and closing cycles. This phenomenon reflects the herb layer’s heightened sensitivity to microhabitat heterogeneity caused by disturbances, such as forest gaps (Jin et al., 2022). The identification of distinct biomass types through cluster analysis offers insights into the adaptive differentiation of understory vegetation across habitat conditions. This analysis provides a scientific foundation for the development of targeted forest management strategies.

### 4.3 Effects of forest structure and environmental factors on understory shrub and herb biomass

This study utilized the SEM methodology to examine the impact of environmental factors and stand structure on the biomass of understory shrubs and herbs in Chinese fir forests. SEM is a statistical method that allows for the simultaneous analysis of complex causal relationships between multiple independent and dependent variables. In addition, it quantifies the magnitude of both direct and indirect pathways. This method has been extensively applied in studies examining the relationship between stand structure and understory vegetation in forest ecosystems (Chen et al., 2025; Wang et al., 2023). A comparison of SEM with traditional regression analysis methods reveals the ability of the former in avoiding estimation biases caused by multicollinearity among variables. This advantage renders SEM more suitable for revealing complex relationships between stand structure and understory vegetation biomass. The findings suggest that stand structure exerts a more significant influence on understory shrub and herb biomass than do environmental factors. Stand structure exerts a negative influence on both shrub biomass (TD-SB and LS-SB), with a greater impact on TD-SB than on LS-SB. This finding is consistent with the conclusion of Barbier et al. (2008) in temperate forests that increased canopy closure inhibits the growth of light-demanding shrubs. The independent effect of Mingling Index on the shrub layer may occur through two mechanisms. On the one hand, competition for water and nutrients among the root systems of different tree species in the topsoil of mixed-species forests may inhibit the growth and nutrient uptake efficiency of shrub roots (Liu et al., 2025); on the other hand, the complex composition of litter in mixed-species forests can alter soil physicochemical properties and microbial activity, thereby indirectly affecting nutrient availability for shrubs (Xiang et al., 2025). The influence of stand structure on understory herb biomass was found to be positive, with the most significant impacts observed for canopy openness and mingling index of tree species. This response direction is incongruent with that of the shrub layer, indicating that herbaceous plants exhibit a greater reliance on diverse microhabitats formed by low light or complex understory structures. This finding aligns with the hypothesis proposed by Chevaux et al. (2022) that stand structure affects herbaceous vascular plants under the canopy more than other plant groups. The presence of high-density shrub layers has been demonstrated to result in a reduction of herbaceous species diversity, a phenomenon attributed to the observed shading effects (Li et al., 2020). Among these factors, canopy openness exhibited the strongest influence on understory vegetation biomass. This finding is consistent with the conclusion of Ahmad et al. (2019) that “forest stand spatial structure significantly impacts understory vegetation biomass”. Competition among plants for resources such as light, nutrients, space, soil, and water (Anderson et al., 2001; Sigurdsson et al., 2005) has been shown to intensify with increasing canopy structure indices, which typically reduce understory biomass (Sabo et al., 2009). Reduced canopy openness limit available light resources for plants, thereby constraining shrub biomass accumulation. Furthermore, the degree of tree size differentiation (Gini coefficient) significantly influences the herb biomass, indicating that enhancing structural heterogeneity is a key strategy for improving multispecies biodiversity in forest management (Uhl et al., 2024).

Among environmental factors, the impact of slope position, slope gradient, human disturbance intensity, and humus thickness on understory vegetation biomass decreased in that order. This finding suggests that, in addition to topographical influences, moderate disturbance may promote understory biomass accumulation by creating forest gaps and facilitating regeneration. This result is consistent with the conclusions of Ren et al. (2021), who reported that forest gaps can reduce resource competition while enhancing resource complementarity and niche effects. However, the influence of humus thickness was found to be relatively minor in this study; this differs from the findings of John et al. (2007), who emphasized that soil nutrients drive the species composition of tropical forests. This discrepancy may be attributed to the relatively simple structure of the subtropical planted Chinese fir forests studied, where understory regeneration is more strongly regulated by stand structure (e.g., canopy closure) than by subtle soil variations. Similarly, the relationship between soil properties and understory vegetation is modulated by the overall fertility background (Baraloto et al., 2011; Chen et al., 2025), and under low-nutrient adaptation or non-limiting conditions, the influence of humus thickness may be less pronounced. The present study found that slope position had a negative effect on MD-HB, suggesting that in specific stand structures and competitive environments, downslope areas experience an accumulation of soil moisture and nutrients due to topographic convergence, which alters the redistribution patterns of light, moisture, and nutrients under the forest canopy, thereby placing stress on the growth of MD-HB (Huang et al., 2015). From the perspective of niche differentiation, the MD-HB may occupy a transitional microhabitat with a narrow tolerance threshold; it has a narrow ecological range regarding water and heat conditions. When changes in slope position trigger the redistribution of soil moisture and nutrients, this type exhibits a negative response due to its limited capacity to buffer environmental fluctuations (Guo et al., 2019). So downslope positions may experience waterlogging or competition from other trees, which imposes stress on the growth of certain medium-density herb types (Christopher et al., 2018). Overall, the impact of environmental factors on the biomass of understory is primarily achieved indirectly through the regulation of stand structure, rather by direct action, indicating that stand structure serves as a key mediating link between environmental factors and understory vegetation (Zheng et al., 2025).

### 4.4 Limitations and shortcomings of the research

The present study is not without its limitations. First, the extant research indicates that the selection of stand structure indicators should be tailored to specific forest types and successional stages (John et al., 2007). The present study primarily validated the applicability of six stand structure indicators in subtropical plantation forests dominated by Chinese fir forests and containing multiple co-dominant tree species. For natural forests or mixed forests with more complex species composition and structure, these indicators may require further adjustment or supplementary validation. Second, the K-means clustering method, which was employed to analyze biomass categories for shrubs and herb biomass separately, does not directly quantifiable in terms of the overall effect of environmental factors and stand structure on understory vegetation biomass. Third, the environmental factors considered did not account for other potential influencing factors, such as the physicochemical properties of the soil (e.g., nitrogen and phosphorus content, pH) and microbial activity; these omitted factors may also exert certain non-negligible effects on understory vegetation biomass.

## 5 Conclusion

This study is pioneering in its integration of cluster analysis (K-means) with the SEM framework, providing a systematic approach to elucidate the differential driving mechanisms of understory shrub and herb biomass in subtropical Chinese fir forests. The findings indicate that modifying or improving stand structure to regulate understory vegetation is imperative for the effective management and sustainable operation of planted forests. The results indicate that understory shrub and herb vegetation is not a homogeneous entity; shrub and herb layer biomass exhibits significantly different responses to the same drivers. Cluster analysis further reveals that even within the same shrub and herb vegetation layer, biomass of different functional types within the shrub and herb layers demonstrates distinct response patterns. In summary, this study applies a quantitative model of multifactorial influences to classify and analyze the biomass of various shrub and herb vegetation types. These analyses contribute to the scientific understanding of the mechanisms that govern understory shrub and herb biomass formation. The findings provide a scientific basis for the near-natural management of subtropical plantations. In the future, forest management practices could implement precise control of understory vegetation by adjusting factors such as canopy openness and species diversity, thereby promoting the health and stability of forest ecosystems. Subsequent research endeavors may encompass a range of forest types and environmental conditions to substantiate the universality and applicability of these findings.

## Data Availability

Since the original data from this study is currently supporting other ongoing projects in our laboratory and remains under internal confidentiality, we are unable to make it publicly available at this time. Requests to access these datasets should be directed to Duoyi Zhang, dy020419@163.com.
